# Alterations in biceps femoris long head fascicle length, Eccentric hamstring strength qualities and single-leg hop distance throughout the ninety minutes of TSAFT^90^ simulated football match

**DOI:** 10.1371/journal.pone.0278222

**Published:** 2022-12-09

**Authors:** Gokhan Yagiz, Vinay Kumar Dayala, Kevin Williams, Julian Andrew Owen, Hans-Peter Kubis

**Affiliations:** Institute for Applied Human Physiology, School of Human and Behavioural Sciences, Bangor University, Bangor, United Kingdom; University of Study of Bari Aldo Moro, ITALY

## Abstract

**Introduction:**

Football matches show higher hamstring strain injuries (HSIs) than football training. The occurrence of HSIs increases in the last fifteen minutes of both halves of football matches and shows an incremental trend towards the end of the ninety minutes.

**Objectives:**

This study aimed to examine football-specific fatigue-induced alterations in risk factors of the HSIs, including biceps femoris long head fascicle length via ultrasonography (BFlh FL), single-leg hop distance, hamstrings’ maximal eccentric strength, and single-leg hamstring bridge test (SLHB) performance.

**Methodology:**

During ninety minutes of the TSAFT^90^ football simulation, the BFlh FL and single-leg hop distance were measured three times (before, at half-time and after 90 minutes of simulated match-play), and maximal hamstrings eccentric strength and SLHB test scores were recorded twice (before and after simulated match-play) for both legs in physically active participants (n = 15).

**Results:**

Maximal eccentric hamstrings’ strength (dominant leg (D): p < 0.001, Hedges’ (adjusted) *g* effect size = -0.969; non-dominant leg (ND): p < 0.001, *g* = -0.929) and the SLHB performance (D: p < 0.001, *g* = -1.249; ND: p < 0.001, *g* = -1.108) showed large decrements immediately after the TSAFT^90^ intervention. There were no significant alterations in the BFlh FL, and the single-leg hop distance.

**Conclusions:**

Maximal eccentric strength and the SLHB performance of hamstrings are reduced after 90 minutes of simulated football match-play. Practitioners may consider focusing on improving eccentric strength and the SLHB performance. Future studies should examine alterations in the BFlh fascicles’ dynamic lengthening and shortening ability during a football match.

## 1. Introduction

Hamstring strain injuries (HSIs) are the most common non-contact injuries in football, representing 12% of all injuries [1]. The typical prevalence of HSI is reported to be in the region of 5 to 6 injuries per season in a football team composed of 25 players [2]. Additionally, hamstring strain re-injuries are higher (16%) [1], more severe, and cause greater time loss than the initial HSIs in football [3]. Moreover, HSIs in football have shown a 4.1% annual increase [4] despite scientists’ increasing efforts to provide an optimal injury prevention method in the last two decades.

Match-caused HSIs show a higher incidence than training-caused HSIs in football (respectively 3.70 (3.43–3.99) vs 0.43 (0.39–0.47) per 1000 hours) [5]. Match-induced HSIs are more frequent in the last fifteen minutes of each half of football matches [1,6,7]. Suggestive that increased muscular fatigue might play a substantial role in multifactorial causations of the HSIs in football [8,9]. From this viewpoint, studies [10–31] focused on exploring interrelationships between ninety minutes of football match-induced fatigue and modifiable risk factors of HSIs are warranted.

Researchers have identified various risk factors for hamstring strain injuries, which were previously divided into modifiable and non-modifiable risk factors [32–34]. Non-modifiable risk factors of HSIs include older age [35–40] and previous lower extremity injuries [40–44]. Modifiable risk factors include, but are not restricted to, decreased eccentric hamstring strength qualities [45–48], lower single-leg hop distance (SLHD) [47], and structural risk factors of hamstrings [33] (shorter biceps femoris long head fascicle lengths (BFlh FL) [40], higher hamstring stiffness [49]).

The vast majority of the previous studies [10–20,22–24,27–29,31] investigating a 90-minute football match or simulated-football match-induced changes in hamstring strength used isokinetic strength assessments except for one study [10], which used a Nordic hamstring exercise device for assessing hamstrings eccentric strength. However, the most recent meta-analysis recalibrating risk factors for HSIs suggested that isokinetic strength values were not a risk factor for future HSIs [33]. Additionally, the meta-analytic evidence [33] also suggests that eccentric strength assessments using Nordic hamstring devices are unrelated to future HSIs. Consequently, studies investigating the immediate effects of 90-minute football or simulated football match on the hamstring strength parameters did not use the specific strength assessments associated with risks for future for HSIs.

Conversely, the single-leg hamstring bridge test (SLHB), which assesses the capacity of repetitive high-force production of the hamstrings [46], and eccentric hamstring strength assessed via a handheld dynamometer [47], has been shown to be associated with initial HSIs [33]. To date, no study examining the effect of soccer match-play on HSI risk has utilised this test for evaluating football match-induced changes in the SLHB performance, and eccentric hamstring strength exists. In addition, no study has investigated the immediate effects of a 90-minute actual or simulated football match on BFlh FL and SLHD, which are significantly associated with future HSIs [33]. Previously, a shorter passive BFlh FL was defined as an independent risk factor for future HSIs by increasing the injury risk more than fourfold (risk ratio: 4.1) [40]. Similarly, a lower SLHD score was defined as a risk factor for future HSIs (odds ratio: 0.884) [47].

Many researchers have adopted simulated soccer match protocols when examining changes in modifiable HSI risk factors during and immediately after soccer match-play due to the problems associated with measurement during actual match-play. A recent systematic review examining the efficacy of soccer match-play simulations concluded that these simulations do not precisely represent the biochemical strains of an actual football match [50]. In response to these findings, da Silva and Lovell [51] designed and validated a 90-minute soccer-specific aerobic field test (T-SAFT^90^), which mimics the mechanical and physiological, immune, endocrine and muscle damage responses of an actual 90-minutes football match. However, no study has adopted the T-SAFT^90^-when examining alterations in risk factors of HSIs.

Therefore, investigating the immediate effects of ninety minutes of the TSAFT^90^ generated fatigue-induced alterations in the risk factors for hamstring strain injuries might bring new insights for improving post-match recovery strategies and preparing optimal injury prevention programs for hamstring strains for football players. Therefore, this study aimed to explore the immediate alterations in the modifiable risk factors of HSIs [33] after TSAFT^90^ by measuring the SLHB performance, eccentric hamstring strength via handheld dynamometer, BFlh FL, and SLHD [40,46,47]. Additionally, the mean percentage of maximal heart rate (%HRmax) for every fifteen minutes of the 90-minute TSAFT^90^ simulated football match was measured as a secondary measurement.

The late-swing phase of running was pointed out as the most vulnerable time of the hamstring muscles [52–54]. At the late-swing phase of running, hamstrings eccentrically contract to decelerate the tibia and to control the antagonist quadriceps femoris muscles’ concentric force [55]. The BFlh reaches 110% of its length at the late swing phase of running [56]. The HSIs most commonly occur when the muscle fascicles cannot resist an excessive elongation during the late-swing phase of running [57]. Therefore, shorter BFlh fascicles [40] and insufficient eccentric hamstring contractions were considered risk factors for HSIs [40,52,53].

It has recently been revealed the BFlh fascicles actively lengthen during eccentric contraction [58]. Additionally, it has previously been pointed out that hamstrings undergo elongations with eccentric contraction during the late-swing phase of running [56]. During this time, an excessive antagonist force higher than the eccentric force of the hamstrings elongates the hamstrings and can lead to damage and strains in BFlh fascicles [40,52,53]. Accordingly, a shorter BFlh fascicle length was defined as a risk factor for HSIs because of a possible lesser ability to be stretched and possible greater damage due to lesser sarcomeres in the series of the BFlh fascicles than longer fascicles during the eccentric muscle activation of the hamstrings [40]. A football match includes around 1687 metres of running and 170 metres of sprinting [59]. Based on these, the present study hypothesised that BFlh FL would increase after each half of the 90-minute TSAFT^90^ simulated football match due to exposure due to the repetitive eccentric elongation and a possible eccentric overload in the hamstrings muscles during a football match that may lead to potential damage in the hamstrings’ muscle fascicles [40], which could negatively affect the shortening ability of the BFlh fascicles could lead increments in the length of the fascicles.

Regarding eccentric hamstring strength, previous studies either used a Nordic hamstring exercise execution device [10] or an isokinetic device [11,14,19,20,29] to assess eccentric hamstring strength reported significant decreases after a football match intervention. Accordingly, the present research hypotheses that maximal eccentric strength and SLHB performance will significantly decrease after the simulated football match. Similarly, single-leg hop distance will decrease immediately after halftime and the full-time 90-minute TSAFT^90^ simulated football match due to fatigue-led strength decrements. Lastly, the %HRmax for every 15 minutes of simulated football intervention will increase throughout the time points.

## 2. Methodology

### 2.1. Study design

A quasi-experimental one-group repeated measures study design was used in the present study. Ethical approval was obtained from the local ethics committee of the School of Human and Behavioural Sciences at Bangor University (code: 2022–17105) according to the declaration of Helsinki (World Medical Association, 2013). Additionally, the individual pictured in Figs 2–4 has provided written informed consent (as outlined in PLOS consent form) to publish their image alongside the manuscript.

**Fig 1:**
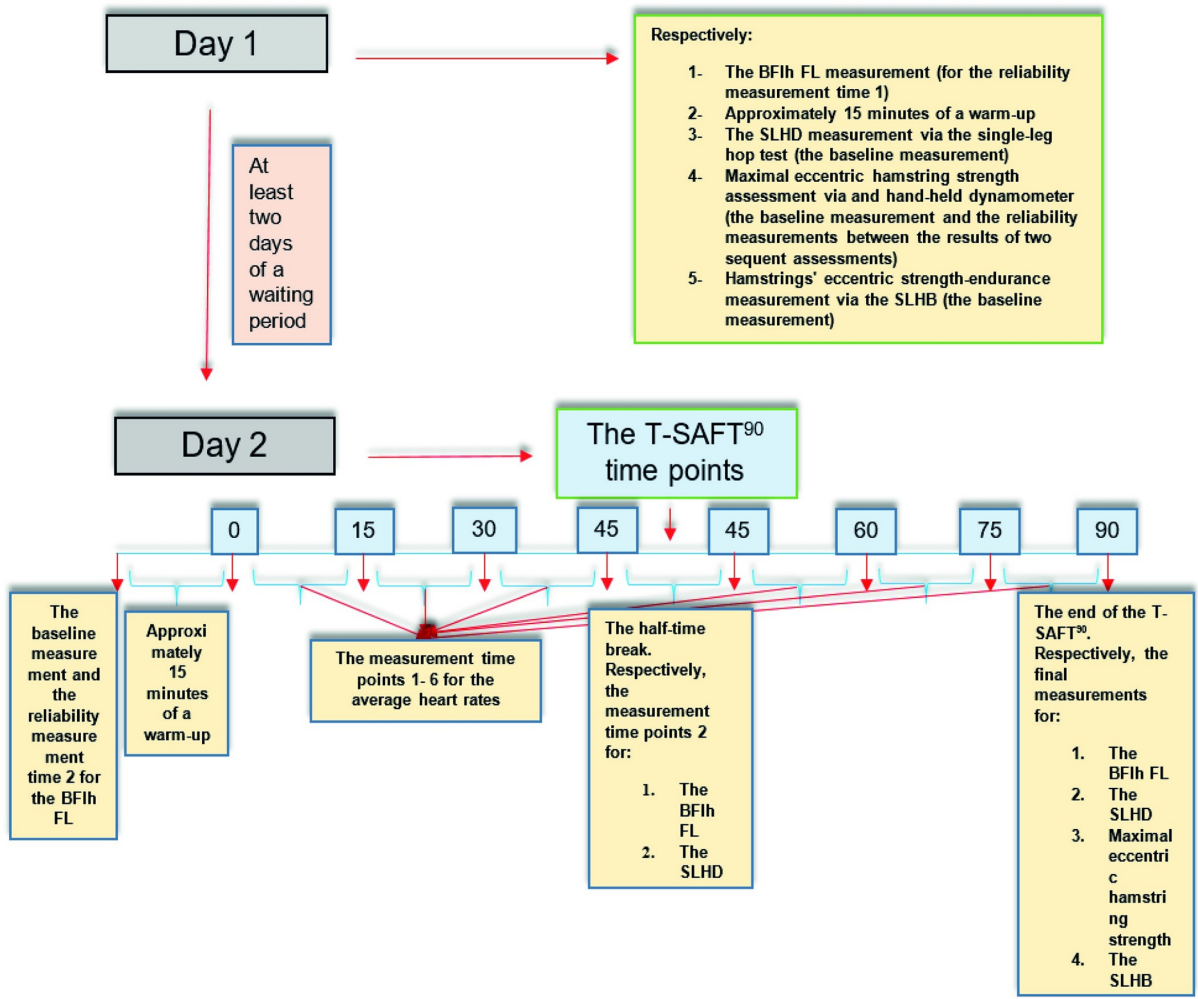
Study flow diagram. This diagram shows the actions taken in the first and second sessions of the study.

On the first day, the first BFlh FL measurements of the reliability assessments, approximately fifteen minutes of a warm-up, baseline measurements of SLHD via single-leg hop test, eccentric maximal hamstrings’ strength via handheld dynamometer, and SLHB tests were completed for both thighs based on the given order in Fig 1. After at least two days of waiting, the TSAFT^90^ intervention was applied to the participants. Most recently, Bueno et al. [10] measured maximal eccentric hamstring strength with 24 hours separations between pre-test and post-test after a football match. However, the present study allowed a longer time (at least 2 days, mean: 5 days) to eliminate possible negative effects of the strength measurements on the post-test. Moreover, the soreness status of the hamstrings was requested from the participants before the second-day measurements, and if the participant mentioned any soreness in the hamstrings, the second-day measurement was postponed to a different day until the participant mentioned a full self-reported recovery of the hamstrings.

During the intervention day, baseline measurements of the BFlh FL for both thighs (which were also second measurements for inter-day intra-rater reliability assessments), approximately fifteen minutes of a warm-up, and ninety minutes of the TSAFT^90^ football simulations were implemented corresponding to the order given in Fig 1. At the half-time of the TSAFT^90^, the BFlh FL and the SLHD were respectively measured for the second time for both thighs (Fig 1). After the ninety minutes of the TSAFT^90^ intervention, the BFlh FL (third and final measurement), the SLHD (third and last measurement), eccentric maximal hamstring strength (second and final measurement), and the SLHB (second and final measurement) were measured for both thighs based on the order given in Fig 1. For detecting the immediate effects of the simulated football match, all the second-day measurements were completed less than five minutes after half-time and less than ten minutes after full-time, according to the order given in Fig 1. Additionally, the %HRmax of each 15 minutes of the 90 minutes of football simulation was calculated. The tests were completed by the first author (GY), an experienced sports physiotherapist in the use of ultrasonography for muscle structure assessments, strength measurements via handheld dynamometry, and SLHB test measurements.

### 2.2. Sample size

The required sample size for this study was calculated using G*Power software version 3.1.9.7 [60]. Effects size (ES = 1.10) was referred from a recent study [10] that investigated the effects of a 90-minutes soccer match on eccentric hamstring strength. However, to ensure the required sample size, the present study chose a 0.5 effect size during the sample size calculation. Additionally, the following parameters were utilised during the sample size calculation: 0.05 alpha level, 0.80 power, one group, two measurement points, 0.5 correlation among repeated measurements and 1 epsilon value that represents the level of sphericity and accepted as 1 for one group repeated measures design. As a result, the required quantity of the sample size was calculated as ten participants. However, this study aimed to recruit at least fifteen participants to increase statistical power.

Reliability studies require a different sample size calculation from the one group repeated measures design sample size calculation above. The required sample size was calculated in light of the intraclass correlation (ICC) value (biceps femoris fascicle length = 0.98 [61]) for the manual linear extrapolation method (MLE), and the lowest single measure ICC (SIMC = 0.837 [47]) value for the same methodology with this study for the maximal eccentric hamstring strength measurement via handheld dynamometry. However, this study chose an ICC value of 0.8 to ensure an adequate sample size for the reliability study. Afterwards, the required sample size was calculated as 7 for two measurements, 0.05 alpha level and 0.80 power, by following the guideline of Bujang and Baharum [62]. However, this study aimed to measure all fifteen participants to minimise the adverse effects of possible dropouts or measurement errors.

### 2.3. Participants

Physically active male participants were recruited via advertisements, e-mail and verbal announcements. Inclusion criteria were considered as a) being male, physically active, healthy and habitually performing at least 75 minutes of vigorous-intensity or 150 minutes of moderate-intensity exercise per week [63], which was assessed via the International Physical Activity Questionnaire short form (IPAQ-sf) [64], b) being free from an acute lower extremity injury, c) being at least 18 years old and maximum 39 years old.

The intervention and testing procedures were verbally explained to the participants before the intervention, and written informed consent was provided to participants on the intervention day. Participants were asked to fill and sign the required forms, e.g. questionnaires and informed consent, before the study. Moreover, participants were advised not to perform exhaustive exercises 48 hours before the TSAFT^90^ intervention and tests [29].

### 2.4. Simulated soccer match protocol (TSAFT^90^)

da Silva and Lovell [51] recently designed the TSAFT^90^ soccer simulation, which includes technical and jumping activities as an addition to the SAFT^90^, and they validated that TSAFT^90^ mimics mechanical and physiological, immune, endocrine and muscle damage responses of a 90-minutes soccer match. The additional technical activities of TSAFT^90^ include passes, shoots, and ball drillings [51]. The T-SAFT^90^ consists of six random and intermittent activities within a 15 minutes period completed three times for each of two 45-minute halves and separated by 15 minutes of a passive resting period, representing a 90-minute soccer match [65]. Performing the activities and arranging the intensity of the activities were maintained via an audio file containing verbal signals obtained from da Silva and Lovell [51].

### 2.5. Warm-up

Approximately fifteen minutes of a warm-up program was performed after BFlh FL measurements on both study days (Fig 1). This warm-up program was completed on a 20-m shuttle on a football pitch. It consisted of twelve football-related exercises: light jogging, side stepping, backward jogging, forward and backwards skipping with arm circles, jumping jacks, high kicks, high knees, dynamic hamstring stretching, walking lunges, sprint, and high knees at higher speeds.

### 2.6. Testing procedures

Before starting the assessments, participants’ height (cm) and body mass (kg) were recorded. The preferred leg for kicking the ball was accepted as the dominant leg. The study procedures were explained verbally, visually and in writing to the participants before the baseline tests and intervention. The testing order and times are defined in the study design section and illustrated in Fig 1. The sequencing of the legs was left to right during all the measurements.

To increase commitment during the single-leg hop test, eccentric maximal hamstrings’ strength measurement and the SLHB test, it was announced to the participants before the tests that certain cash prizes would be given to the first three average scores of the pre- mid- and post-tests of both legs.

#### 2.6.1. Biceps femoris long head fascicle length measurement

**Fig 2:**
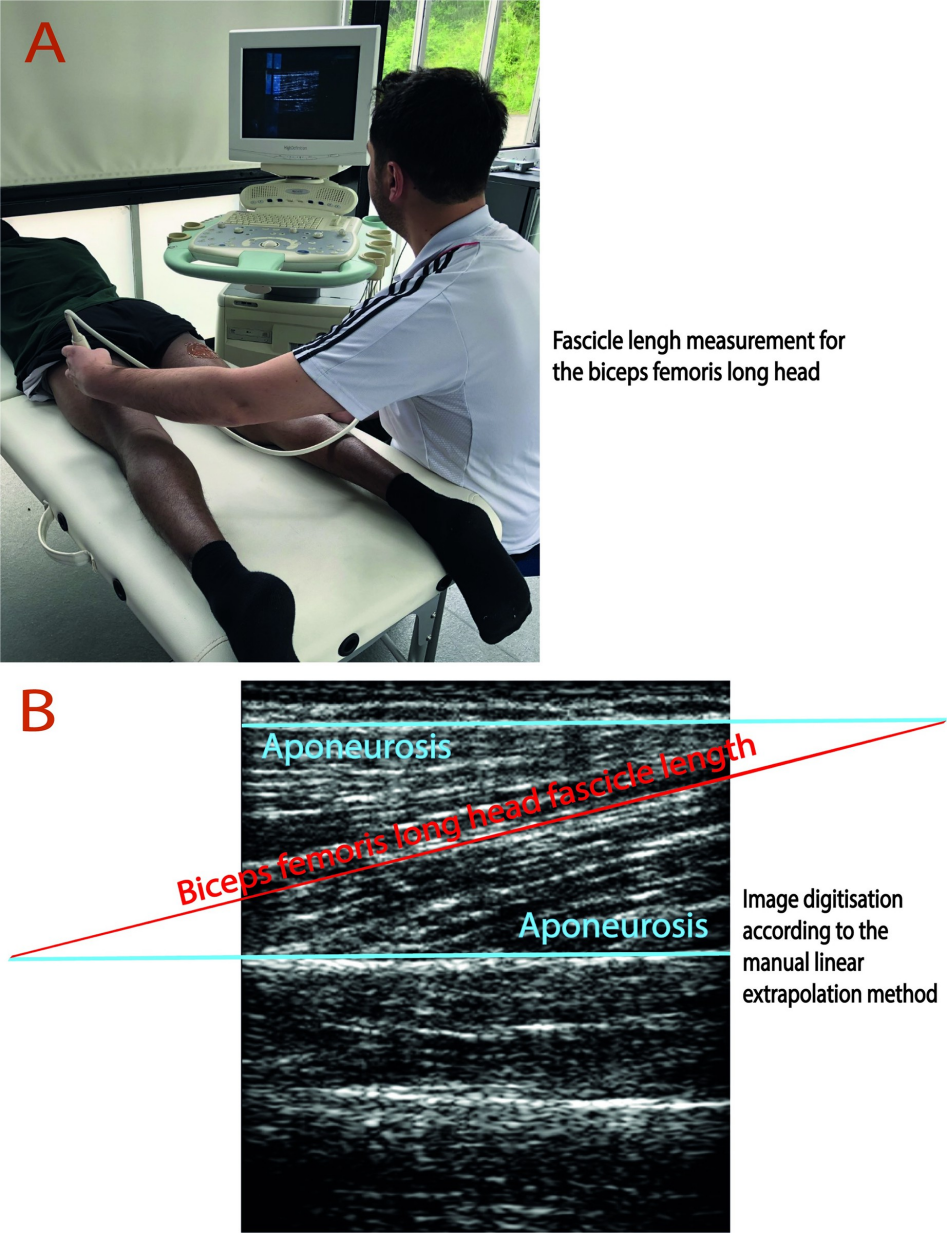
The biceps femoris long head fascicle length measurement and digitisation. A: 2-dimensional ultrasound images were taken from the mid-thigh for both legs. B: Digitisation for the biceps femoris long head fascicle length according to the manual linear extrapolation method. The individual pictured in Fig 2 has provided written informed consent (as outlined in PLOS consent form) to publish their image alongside the manuscript.

A two-dimensional B-mode ultrasound (US) (Esaote, MyLab 50, the Esaote Group, Genova, Italy) was used to measure the BFlh FL for the dominant and non-dominant limbs of the participants. Participants laid prone on a standard medical bed, as shown in Fig 2A [66], and were asked not to perform any voluntary muscle contractions during the measurements. Two US images of the BFlh FL were taken from the mid-point distance between the popliteal crease and the trochanter major when the BFlh was passive [40,66] (Fig 2A). Previously, the passive BFlh FL was defined as a risk factor for HSIs [40]. Therefore, this study measured the BFlh FL without voluntary contraction, namely at a passive position, as previously described [40].

US measurement was performed using a linear array ultrasound probe (LA523E, 7.5–12 MHz, the field of view: 5 cm depth x 4.7 cm width) (Fig 2A). During the measurements, a minimum pressure was applied to minimise the possible effects of the pressure on the BFlh FL measurements [67]. Firstly, the US probe was placed transverse to the BFlh to monitor the cross-sectional area of the BFlh. After ensuring the correctness of the location of the BFlh, the US probe was turned in a parallel orientation with the BFlh muscle orientation. At this position, slight ultrasound probe adjustments were applied to visualise the aponeuroses. Then, two longitudinal BFlh muscle architecture images were taken (Fig 2A). The mean values for the BFlh FL of these two images were calculated as the fascicle length [68]. External markers (e.g. scars, freckles and the distance of the features to the measurement points) [69] and internal markers (subcutaneous adipose tissue and markers between fascicles) [70] of the first US measurements were referred to ensure the reproducibility and correctness of the measurement places of the BFlh FL in the sequent assessment time points.

The ImageJ software (ImageJ, National Institutes of Health, Bethesda, Maryland, USA) was used for calculating the length of the BFlh FL by using the MLE method described by Potier et al. [68]. Before the ImageJ calculations, the architectural features of the BFlh were drawn using Adobe Illustrator software, which was also previously used for scientific digitisations [71,72], according to the MLE method [68]. The superficial and intermediate aponeuroses were drawn and extended over their visible lengths (Fig 2B). Then, the visible part of the BFlh FL was drawn and extended until reaching the extensions of the aponeuroses (Fig 2B). After setting the scale of the measurement units in the ImageJ software, the BFlh FL was calculated as the mean of the two US pictures for each assessment. Additionally, the BFlh FL measurement reliability assessment was performed for both legs between the BFlh FL measurements in the first session and baseline measurements in the second session.

#### 2.6.2. Eccentric hamstring strength measurement

**Fig 3:**
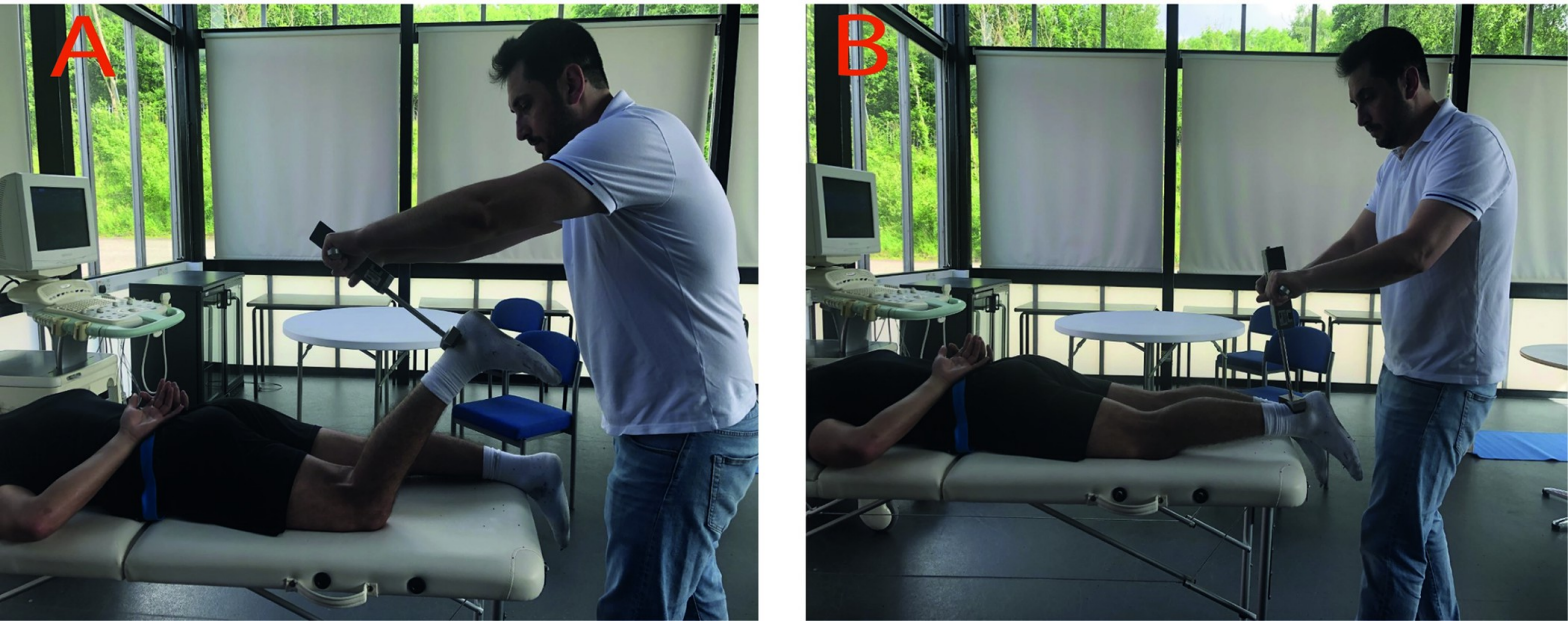
Hamstrings’ eccentric maximum strength measurements by handheld dynamometry. The hip joints were neutral (0 degrees of hip extension) during the measurements. A: The starting point of the measurement was when the knee was approximately 60⁰ flexed and when the handheld dynamometer was on the two centimetres proximal to the malleolus of the ankle. B: The ending position of the hamstrings’ maximum eccentric strength measurement. The individual pictured in Fig 3 has provided written informed consent (as outlined in PLOS consent form) to publish their image alongside the manuscript.

Hamstrings’ maximal eccentric strength was measured using a handheld dynamometer (CSD 300 Strength Dynamometer, Chatillon, Largo, Florida) following the protocol suggested by Goossens et al. [47]. Participants laid down in a prone position on a standard medical bed for the test. The knee-joint angles were measured by using a goniometer. Participants’ legs were positioned at the start position (Fig 3A), and the participants were asked to hold and resist the pressure applied by the assessor via the handheld dynamometer (Fig 3A and 3B). Additionally, participants were informed that the assessor would eventually pull the lower leg down [47] (Fig 3B). For each leg, two measurements were completed, and the highest measure was accepted as the maximal eccentric strength of the hamstrings [47]. Additionally, using the results of the two measurements, intra-tester reliability was assessed. The starting point of the measurement (Fig 3A) and the finishing point of the assessment (Fig 3B) are shown in Fig 3. Each test was finished in about five seconds, and the tester accordingly applied a pressure to allow the participants to produce their maximal eccentric hamstring force in a similar time and similar velocity.

#### 2.6.3. Single-leg hamstring bridge performance

The capacity of repetitive high-force production of the hamstrings was assessed using the SLHB test, as suggested by Freckleton and colleagues [46]. The single-leg hamstring bridge test is a reliable test [73,74] and mimics the functional capacity of the hamstring as similar to the late swing phase of running [46].

**Fig 4:**
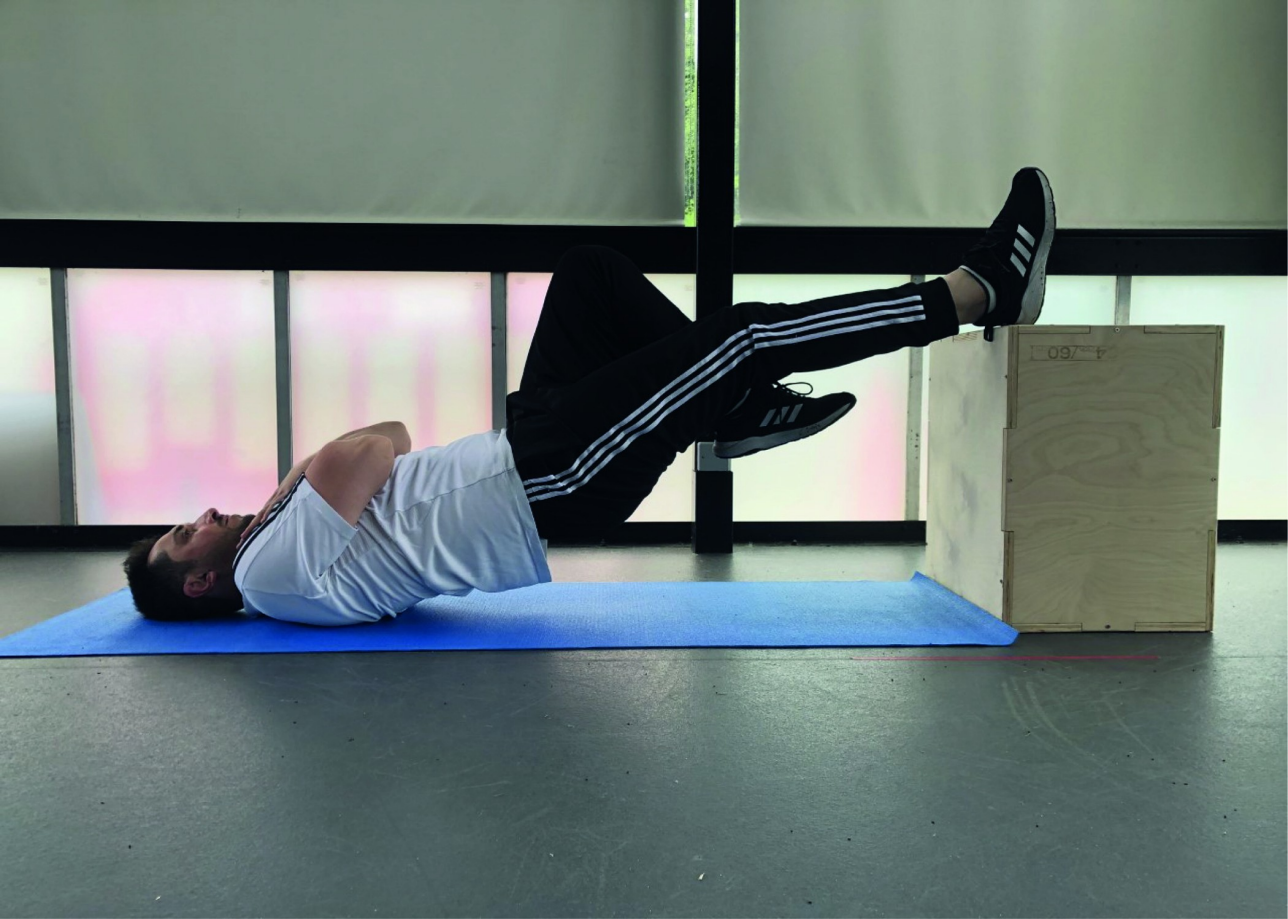
Single Leg Hamstring Bridge (SLHB) test. Participants were asked to perform movements by touching the ground by their bottom and reach 0⁰ of hip extension by lifting their bottom respectively and without resting with the help of a 60-cm high box. The other leg was in a vertical position as inactive and stationary to eliminate any momentums that might be provided by swinging the leg. Participants were advised to aim to perform as much as they could repeat the same movement until failure. The individual pictured in Fig 4 has provided written informed consent (as outlined in PLOS consent form) to publish their image alongside the manuscript.

To perform the single-leg hamstring bridge test, participants laid down on the floor and put one heel on a box at 60 cm height [46]. The arms of the participants were crossed on their chests [46]. The leg, which would be tested, was at around twenty degrees of knee flexion as defined previously [46]. Then, participants pushed down their tested heels and lifted their bottoms off the floor [46]. Participants were asked to maintain the movements by touching the ground by their bottom and reach 0⁰ of hip extension by lifting their bottom without resting [46]. The other leg was in a vertical position as stationary to eliminate any momentums that might be provided by swinging the leg [46]. Participants were advised to aim to do as much as possible to repeat the same movement until failure [46]. Feedback was given consistently to ensure the correct technique’s achievement over the test [46]. In the case of losing the proper form, participants were warned once, and a subsequent fault in the technique led to ceasing the test [46]. Maximum repetitions were recorded as the outcome of the tested leg, and the same test was applied to the other leg [46]. The SLHB testing position has illustrated the Fig 4.

#### 2.6.4. Single-leg hop distance measurement

The SLHD measurements were completed via the single-leg hop test by following the instructions of Goossens et al. [47], who modified the hop test described by Munro and Herrington [75]. Participants performed three successful single-leg jumps as far as possible, maintaining the landing position on the same footprint for three seconds. Subsequently, each leg’s best scores were accepted as the single-leg hop distance [47]. During the test, the usage of arms was not restricted, and participants wore sports shoes [47].

#### 2.6.5. The mean percentage of maximal heart rate

The %HRmax was measured every 15 minutes of the TSAFT^90^ by using a heart rate tracking system (Activio Telemetry Heart Rate System, Activio International AB, Bastad, Sweden).

### 2.7. Statistical analyses

Primary statistical analyses of the results were performed using the SPSS software (IBM Corporation, Chicago, Illinois). Participants’ characteristics (age, height and weight) were given in means and standard deviations. One-way repeated measures ANOVA and Bonferroni posthoc test were employed for analysing the following dependent variables: %HRmax, the BFlh FL, eccentric maximal hamstring strength, the SLHB score and the SLHD variables for one group and between two to six measurement points depending on the variable. The ICC values were calculated for two-way random, absolute agreements for single measures for the reliability analyses [76]. It has been interpreted that an ICC value less than 0.5 indicate poor reliability, an ICC value between 0.5 and 0.75 represents moderate reliability, an ICC value between 0.75 and 0.9 means high reliability, and an ICC value over 0.9 is indicative of very high reliability [77]. Moreover, the Hedges’ (adjusted) *g* effect sizes were automatically calculated for one group repeated measures design by entering the means and SDs of the pre-tests and post-tests, correlations between pre-tests and post-tests, and sample size to the Comprehensive Meta-Analysis software (CMA, version 3.0, Biostat, Englewood, New Jersey) [78]. The main difference between the Hedges’ (adjusted) *g* and the Cohen’s d is the better estimation of the Hedges’ g by adjusting potentially biased estimates than the Cohen’s d for sample sizes smaller than twenty participants [79]. The Hedges’ g effect sizes were interpreted as small (0.2), medium (0.5) or large (0.8) [80].

## 3. Results

Eighteen participants were initially recruited. One participant did not meet the inclusion criteria. Two participants did not attend the second session of the study. In short, seventeen participants attended the first session. However, only fifteen participants completed the study (n = 15, age = 25.73 ± 5.98 years, height = 172.45 ± 5.17 cm, weight = 72.27 ± 7.22 kg). The dominant leg was detected as the right side in all participants. Participants mentioned that they perform weekly 7.14 ± 5.07 hours of vigorous physical activity and 5.18 ± 3.14 years of sports-specific training history (from recreationally active to a professional level: eight participants were football players, two participants were cricket players, one participant was a sprinter, one participant was a boxer, one participant was a kickboxer, one participant was a volleyball player, one participant was a kayaker). None of the participants mentioned a lower extremity injury history.

### 3.1. The mean percentage of maximal heart rate

**Fig 5:**
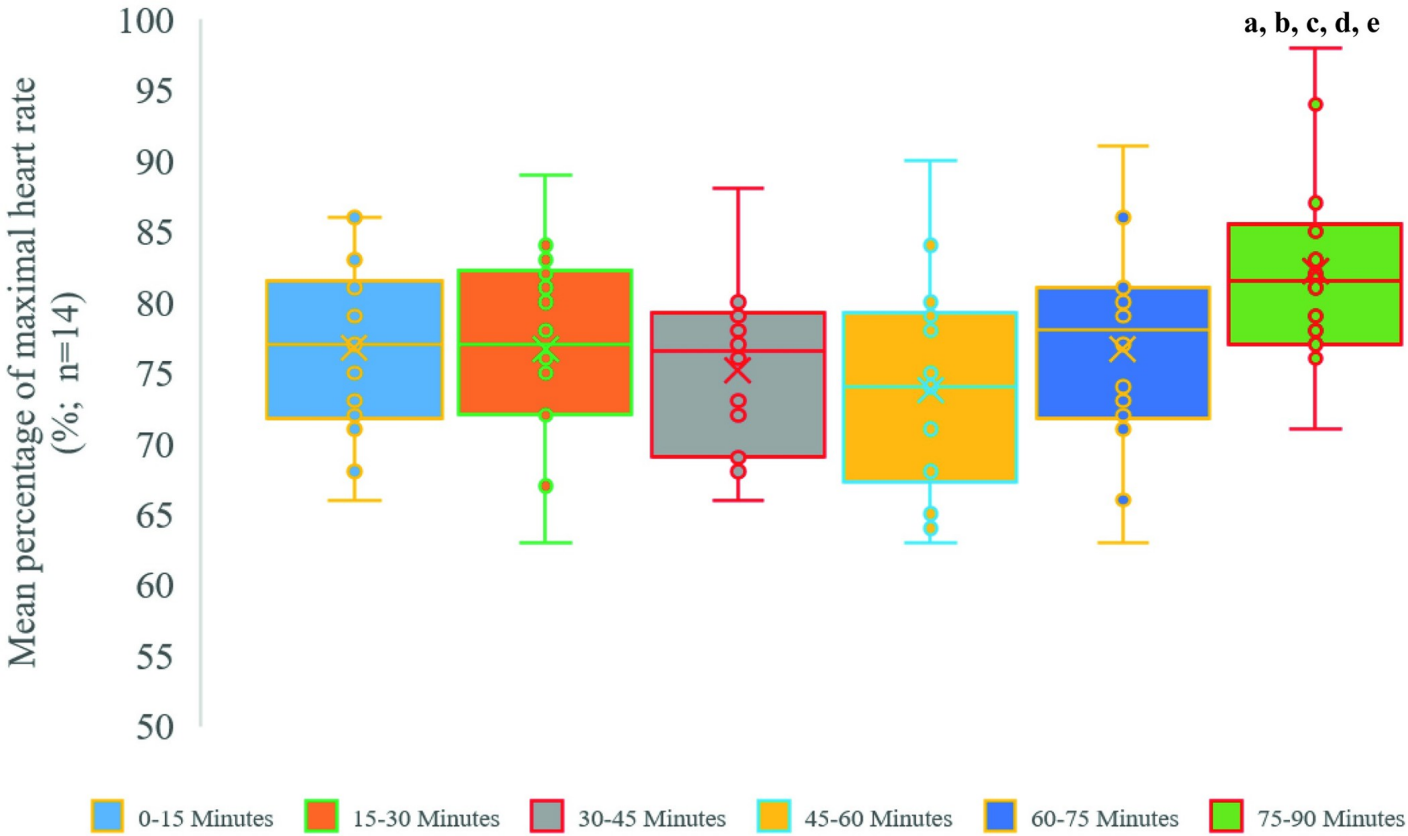
**Box & Whisker plots show the mean percentage of maximal heart rate of each fifteen minutes across ninety minutes of the TSAFT**^**90**^ **football simulation.** The first box with blue colour represents the mean percentage of maximal heart rate of 0–15 minutes, the second box with the orange colour indicates the mean percentage of maximal heart rate of 15–30 minutes, the third box with grey colour refers to the mean percentage of maximal heart rate of the 30–45 minutes, the fourth box with yellow colour points out the mean percentage of maximal heart rate of the 45–60 minutes, the fifth box with navy blue colour demonstrates the mean percentage of maximal heart rate of the 60–75 minutes, and the sixth box with green colour shows the mean percentage of maximal heart rate of the 75–90 minutes of the TSAFT^90^ football simulation. Abbreviations: **a**, significantly higher than 0–15 minutes (p = 0.021); **b**, significantly higher than 15–30 minutes (p = 0.014); **c,** significantly higher than 30–45 minutes (p < 0.001); **d,** significantly higher than 45–60 minutes (p = 0.001); **e**, significantly higher than 60–75 minutes (p = 0.013). Abbreviations: n, sample size.

One participant’s %HRmax measurement was not completed due to a technical error. Therefore, the %HRmax measurements were completed for fourteen participants every fifteen minutes throughout the ninety minutes TSAFT^90^ football simulation. Based on the results, the %HRmax of the last 15 minutes of the TSAFT^90^ was significantly higher than the rest of the time points, and there was no significant difference between the rest of the measurement time points (Fig 5).

### 3.2. Alterations in the biceps femoris long head fascicle length

**Fig 6:**
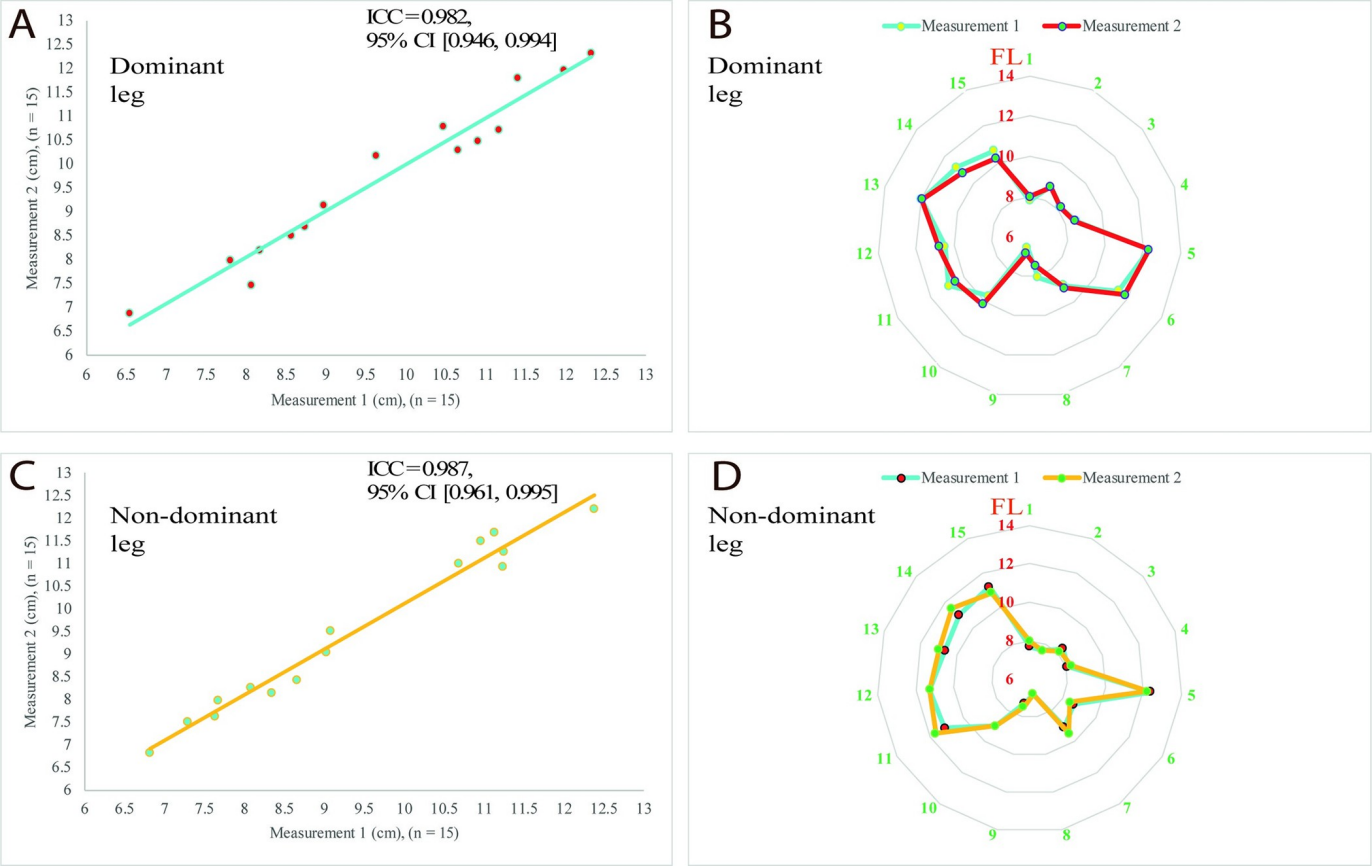
Reliability results of the biceps femoris long head fascicle length measurements for both legs. A: Scatter plots with a regression line for the dominant leg reliability measurements, B: Radar chart for the dominant leg reliability measurements shows agreements between measurements for each participant from 1 to 15, C: Scatter plots with a regression line for the non-dominant leg reliability measurements, D: Radar chart for the non-dominant leg reliability measurements shows agreements between measurements for each participant from 1 to 15. Abbreviations: CI, confidence interval; FL, fascicle length; ICC: Intraclass correlation coefficient; n, sample size.

For both thighs, BFlh FL measurements showed very high reliability results (n = 15, dominant ICC = 0.982, 95% CI [0.946, 0.994], percentage coefficient of variation (CV %) = 2%; non-dominant ICC = 0.987, 95% CI [0.961, 0.995], CV % = 1.7%) (Fig 6). However, no significant differences were detected between measurement time points for both legs’ BFlh FL and the average BFlh FL of the legs due to the ninety minutes TSAFT^90^ simulation (Table 1).

**Table 1:** Alterations in the biceps femoris long head fascicle length (n = 15).

Thigh	Baseline (mean ± SD) (cm)	Half-time (mean ± SD) (cm)	Full-time (mean ± SD) (cm)	Mean ± SD change (half-time vs baseline) (cm)	Mean ± SD change (full-time vs baseline) (cm)	Mean ± SD change (full-time vs half-time) (cm)	p-value (half-time vs baseline)	p-value (full-time vs baseline)	p-value (full-time vs half-time)	Effect size (half-time vs baseline) (Hedges’ (adjusted) *g*)	Effect size (full-time vs baseline) (Hedges’ (adjusted) *g*)	Effect size (full-time vs half-time) (Hedges’ (adjusted) *g*)
**Dominant**	9.69 ± 1.71	9.76 ± 1.49	9.82± 1.75	0.07 ± 0.85	0.13 ± 0.81	0.06 ± 0.94	**p = 1.0**	**p = 1.0**	**p = 1.0**	**0.044** **(trivial)**	**0.07 (trivial)**	**0.03** **(trivial)**
**Non-dominant**	9.46± 1.80	9.53 ± 1.76	9.57 ± 1.65	0.07 ± 0.87	0.11 ± 0.8	0.04 ± 0.97	**p = 1.0**	**p = 1.0**	**p = 1.0**	**0.037** **(trivial)**	**0.058** **(trivial)**	**0.021** **(trivial)**
**Average of both**	9.57 ± 1.66	9.65± 1.4	9.69 ± 1.61	0.08 ± 0.7	0.12 ± 0.63	0.04 ± 0.6	**p = 1.0**	**p = 1.0**	**p = 1.0**	**0.044 (trivial)**	**0.07** **(trivial)**	**0.027** **(trivial)**

**Abbreviations:** n, Sample size; N, Newton; SD, Standard Deviation.

### 3.3. Changes in the maximal eccentric hamstring strength

**Fig 7:**
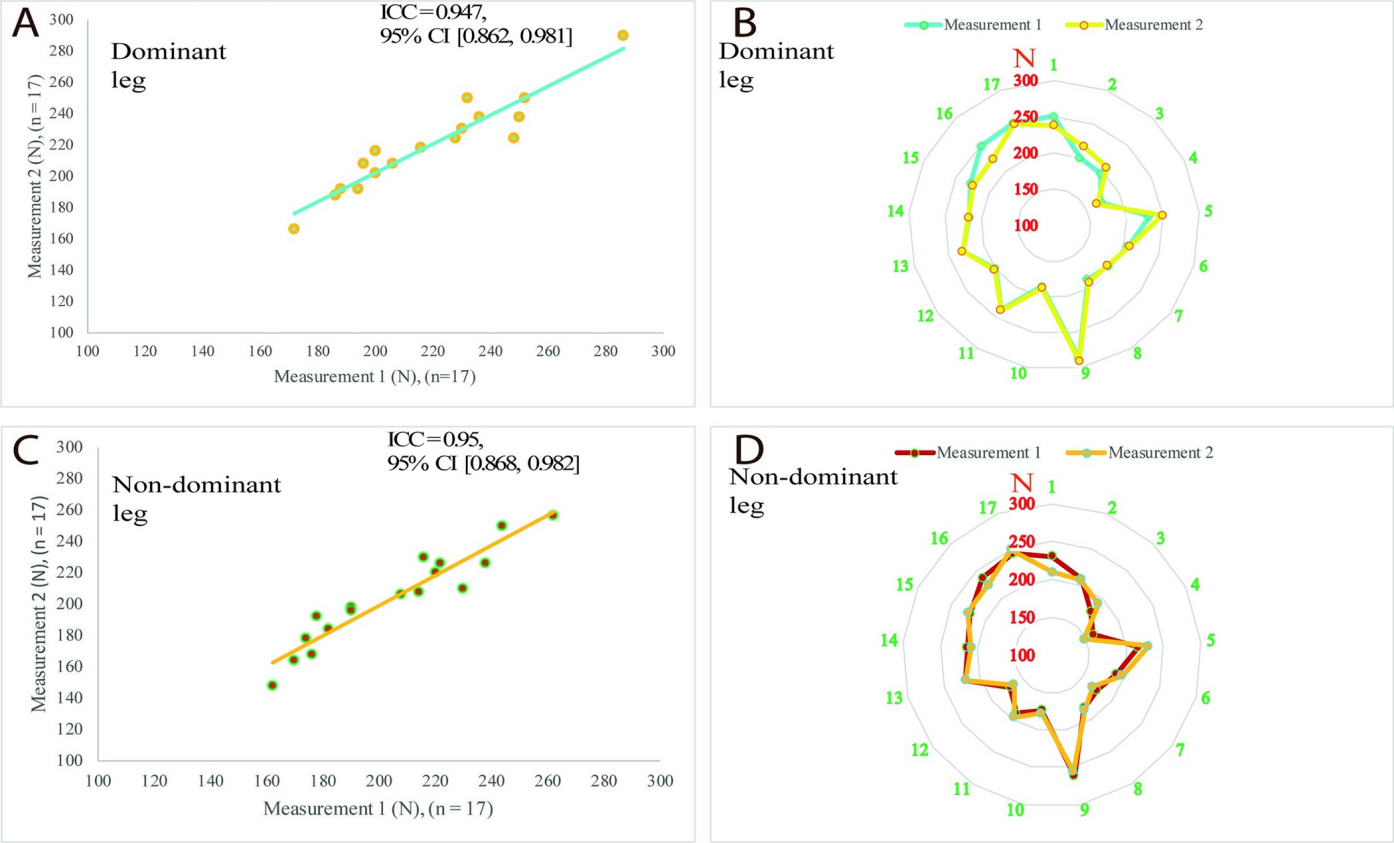
Reliability results from the maximal eccentric hamstring strength measurements for both legs via handheld dynamometry. A: Scatter plots with a regression line for the dominant leg reliability measurements, B: Radar chart for the dominant leg reliability measurements shows agreements between measurements for each participant from 1 to 17, C: Scatter plots with a regression line for the non-dominant leg reliability measurements, D: Radar chart for the non-dominant leg reliability measurements shows agreements between measurements for each participant from 1 to 17. Abbreviations: CI, confidence interval; ICC: Intraclass correlation coefficient; N, Newton; n, sample size.

High to very high reliability results were observed for the maximal eccentric hamstring strength by the handheld dynamometry for both legs (n = 17, dominant ICC = 0.947, 95% CI [0.862, 0.981], CV % = 2.1%; non-dominant ICC = 0.95, 95% CI [0.868, 0.982], CV % = 2.8%) (Fig 7). There were significantly large reductions in the maximal eccentric hamstring strength for both legs and the average of both legs following the 90 minutes TSAFT^90^ simulation (p < 0.001, g = from -0.969 to 0.929) (Table 2).

**Table 2:** Changes in the eccentric maximal hamstring strength were measured by handheld dynamometry (n = 15).

Thigh	Baseline (mean ± SD) (N)	Full-time (mean ± SD) (N)	Mean ± SD change (full-time vs baseline) (N)	p-value	Effect size (full-time vs baseline) (Hedges’ (adjusted) *g*)
**Dominant**	219 ± 25	179 ± 42	-40 ± 31	**p < 0.001**	**-0.969 (large)**
**Non-dominant**	206 ± 25	165 ± 43	-41 ± 30	**p < 0.001**	**-0.929 (large)**
**Average of both**	213 ± 25	172 ± 42	-41 ± 30	**p < 0.001**	**-0.952 (large)**

**Abbreviations:** n, Sample size; N, Newton; SD, Standard Deviation.

### 3.4. Alterations in the single-leg hamstring bridge test performance

There were significantly large reductions in the SLHB performance based on the SLHB scores for both legs and the average of both legs (p < 0.001, g = from -1.249 to -1.108) (Table 3).

**Table 3:** Alterations in the SLHB performance (n = 15).

Thigh	Baseline (mean ± SD) (reps)	Full-time (mean ± SD) (reps)	Mean ± SD change (full-time vs baseline) (reps)	p-value	Effect size (full-time vs baseline) (Hedges’ (adjusted) *g*)
**Dominant**	29 ± 6	20 ± 7	-9 ± 4	**p < 0.001**	**-1.249 (large)**
**Non-dominant**	27 ± 6	19 ± 6	-8 ± 4	**p < 0.001**	**-1.108 (large)**
**Average**	28 ± 6	19 ± 7	-9 ± 4	**p < 0.001**	**-1.193 (large)**

**Abbreviations:** n, Sample size; reps, repetitions; SD, Standard Deviation; SLHB, Single-leg hamstring bridge test.

### 3.5. Alterations in the single-leg hop distance

No significant differences were observed between measurement time points of the SLHD for both legs and the average SLHD results due to the ninety minutes TSAFT^90^ simulation (Table 4).

**Table 4:** Changes in single-leg hop distance (n = 15).

Leg	Baseline (mean ± SD) (cm)	Half-time (mean ± SD) (cm)	Full-time (mean ± SD) (cm)	Mean ± SD change (half-time vs baseline) (cm)	Mean ± SD change (full-time vs baseline) (cm)	Mean ± SD change (full-time vs half-time) (cm)	p-value (half-time vs baseline)	p-value (full-time vs baseline)	p-value (full-time vs half-time)	Effect size (half-time vs baseline) (Hedges’ (adjusted) *g*)	Effect size (full-time vs baseline) (Hedges’ (adjusted) *g*)	Effect size (full-time vs half-time) (Hedges’ (adjusted) *g*)
**Dominant**	184 ± 27.67	187.8 ± 20.33	188.4 ± 22.95	3.8 ± 13.39	4.4 ± 12.68	0.6 ± 7.19	**p = 0.871**	**p = 0.601**	**p = 1.0**	**0.127** **(trivial)**	**0.153 (trivial)**	**0.024** **(trivial)**
**Non-dominant**	180.01 ± 22.59	180.14 ± 23.38	179.17 ± 24.81	0.13 ± 14.81	-0.85 ± 12.45	-0.97± 11.54	**p = 1.0**	**p = 1.0**	**p = 1.0**	**0.005** **(trivial)**	**-0.033** **(trivial)**	**-0.038** **(trivial)**
**Average of both**	182 ± 24.05	183.57 ± 21.37	183.78 ± 22.51	1.57 ± 10.27	1.78 ± 9.61	0.21 ± 7.05	**p = 1.0**	**p = 1.0**	**p = 1.0**	**0.063 (trivial)**	**0.071** **(trivial)**	**0.009** **(trivial)**

**Abbreviations:** n, Sample size; SD, Standard Deviation.

## 4. Discussion

To the authors’ knowledge, this study is the first study that examined changes in the SLHD and the BFlh FL throughout a ninety minutes simulated football match. Additionally, this study differs from the previous simulated football studies in terms of methods of maximal eccentric hamstring strength and the SLHB performance measurements by using predictive methods for HSIs [46,47]. There were no significant alterations in the SLHD and BFlh FL after 45 minutes and 90 minutes of TSAFT^90^. However, the TSAFT^90^ simulated football match led to significantly large decrements in the maximal eccentric strength. Additionally, the mean percentage of maximal heart rate was significantly higher in the last fifteen minutes of the simulated football match than in the rest of the measurement points.

The passive BFlh FL is defined as the architectural risk factor for future HSIs [40]. In addition to this, there was no study that examined alterations in the architectural risk factor of HSIs after ninety minutes of a football match. Therefore, this study aimed to observe whether there would be an alteration in the architectural risk factor of the HSIs or not. It has been stated that BFlh fascicles actively lengthen during eccentric contraction [58]. However, the effects of eccentric training on the BFlh FL are controversial depending on the ultrasound measurement methods [81–84]. This contradiction could be caused by the absence of a gold standard for the BFlh FL measurements [61], which might be a limitation for the present study. Regarding the immediate effects of playing football on the BFlh FL, Gonçalves (2017) [85] examined the influence of a forty-five minutes football simulation (SAFT^45^) on the BFlh FL. They detected no changes in the fascicle length; however, no study has investigated alterations in the BFlh FL following ninety minutes of simulated or an actual football match. Accordingly, this study confirms the findings of Gonçalves (2017) [85] that there are no changes in the BFlh FL after forty-five minutes of a simulated football match and adds that there are no alterations in the BFlh FL after ninety minutes of the TSAFT^90^ football simulation. Future studies can examine the association between football-induced fatigue and BFlh fascicles’ lengthening-shortening ability.

From the perspective of changes in maximal eccentric hamstring strength and hamstrings’ SLHB performance parameters, the present study reported large reductions (*g* = from -0.969 to -0.929) after the ninety minutes of a TSAFT^90^ football simulation. Moreover, this study detected larger reductions in the hamstrings’ strength qualities measured by the SLHB test (*g* = from -1.249 to -1.108) compared with maximal eccentric strength reductions measured via handheld dynamometry (*g* = from -0.969 to -0.929). Similarly, Bueno et al. (2021) [10] have recently observed large decrements in hamstrings’ eccentric strength after a real football match (Cohen’s d = -1.1). The present study confirms the findings of Bueno et al. (2021) [10].

Based on the results of the present study, both hamstrings’ capacity to produce repetitive high force and maximal eccentric hamstrings strength showed large decrements after performing the TSAFT^90^ football simulation. However, decrements in the SLHB scores were relatively higher than the decrease in their maximal eccentric strength. The SLHB is a test that uses a constant external force obtained by a portion of the participant’s body weight and assesses maximum repetitions against the same force. The SLHB test could represent hamstrings’ repetitive high force production capacity rather than the maximal eccentric strength or eccentric peak torque. Improving hamstrings’ repetitive high-force production capacity via the SLHB should be targeted together with improving the hamstrings’ eccentric strength in the vulnerable population of athletes for HSIs.

This study used the MLE method for calculating the BFlh FL. The MLE method did not significantly differ from panoramic ultrasound scanning, while trigonometric equations were significantly overestimating the BFlh FL [61]. Nevertheless, using the MLE method can be considered a limitation of this study due to the absence of a gold standard method for BFlh FL measurements in the literature. Another confounding factor might be not only including professional football players in the study. Despite the high to very high intra-tester reproducibility of maximal eccentric strength measurements of the present study, using a handheld dynamometer can be another limitation because of its user dependence which requires experience and high physical power; these requirements might lead to inter-tester differences. Additionally, the TSAFT^90^ interventions were completed in outdoor conditions, which can add uncountable variability to the results. However, the outcome measurements were completed under indoor conditions in the same room.

## 5. Conclusions

The ninety minutes of the TSAFT^90^ football simulation leads to large decrements in the hamstrings’ maximal eccentric strength and the SLHB performance in both legs. However, the TSAFT^90^ football simulation doesn’t significantly alter the passive mid-BFlh FL and doesn’t alter the single-leg hop distance after half-time and full-time of the match. Therefore, scientists, conditioners, physiotherapists etc., should focus on improving hamstrings’ eccentric strength and repetitive high force production ability via the SLHB. Future studies can examine changes in lengthening-shortening abilities of the BFlh FL during a ninety minutes football match to bring insights into the prevention strategies of the HSIs.

## Supporting information

S1 File Raw data. (PDF)

## References

[1] Ekstrand J, Hägglund M, Waldén M. Injury incidence and injury patterns in professional football: the UEFA injury study. Br J Sports Med. 2011;45(7):553–8. doi: 10.1136/bjsm.2009.060582 19553225

[2] Ekstrand J, Hägglund M, Kristenson K, Magnusson H, Waldén M. Fewer ligament injuries but no preventive effect on muscle injuries and severe injuries: an 11-year follow-up of the UEFA Champions League injury study. Br J Sports Med. 2013;47(12):732–7. doi: 10.1136/bjsports-2013-092394 23813543

[3] Ekstrand J, Krutsch W, Spreco A, van Zoest W, Roberts C, Meyer T et al. Time before return to play for the most common injuries in professional football: a 16-year follow-up of the UEFA Elite Club Injury Study. British Journal of Sports Medicine. 2020;54(7):421–6. doi: 10.1136/bjsports-2019-100666 31182429 PMC7146935

[4] Ekstrand J, Waldén M, Hägglund M. Hamstring injuries have increased by 4% annually in men's professional football, since 2001: a 13-year longitudinal analysis of the UEFA Elite Club injury study. British Journal of Sports Medicine. 2016; 50(12):731. doi: 10.1136/bjsports-2015-095359 26746908

[5] Ekstrand J, Hägglund M, Waldén M. Epidemiology of Muscle Injuries in Professional Football (Soccer). The American Journal of Sports Medicine. 2011;39(6):1226–32. doi: 10.1177/0363546510395879 21335353

[6] Hawkins RD, Hulse MA, Wilkinson C, Hodson A, Gibson M. The association football medical research programme: an audit of injuries in professional football. Br J Sports Med. 2001;35(1):43–7. doi: 10.1136/bjsm.35.1.43 11157461 PMC1724279

[7] Woods C, Hawkins RD, Maltby S, Hulse M, Thomas A, Hodson A. The Football Association Medical Research Programme: an audit of injuries in professional football—analysis of hamstring injuries. Br J Sports Med. 2004;38(1):36–41. doi: 10.1136/bjsm.2002.002352 14751943 PMC1724733

[8] McCall A, Carling C, Nedelec M, Davison M, Le Gall F, Berthoin S et al. Risk factors, testing and preventative strategies for non-contact injuries in professional football: current perceptions and practices of 44 teams from various premier leagues. Br J Sports Med. 2014;48(18):1352–7. doi: 10.1136/bjsports-2014-093439 24837243

[9] Meurer MC, Silva MF, Baroni BM. Strategies for injury prevention in Brazilian football: Perceptions of physiotherapists and practices of premier league teams. Phys Ther Sport. 2017;28:1–8. doi: 10.1016/j.ptsp.2017.07.004 28886473

[10] Bueno CA, de Araujo Ribeiro-Alvares JB, Oliveira GdS, Grazioli R, Veeck F, Pinto RS et al. Post-match recovery of eccentric knee flexor strength in male professional football players. Physical Therapy in Sport. 2021;47:140–6. doi: 10.1016/j.ptsp.2020.11.032 33279801

[11] Cohen DD, Zhao B, Okwera B, Matthews MJ, Delextrat A. Angle-specific eccentric hamstring fatigue after simulated soccer. Int J Sports Physiol Perform. 2015;10(3):325–31. doi: 10.1123/ijspp.2014-0088 25203540

[12] Coratella G, Bellin G, Beato M, Schena F. Fatigue affects peak joint torque angle in hamstrings but not in quadriceps. J Sports Sci. 2015;33(12):1276–82. doi: 10.1080/02640414.2014.986185 25517892

[13] de Abreu Camarda SR, Denadai BS. Does muscle imbalance affect fatigue after soccer specific intermittent protocol? J Sci Med Sport. 2012;15(4):355–60. doi: 10.1016/j.jsams.2011.11.257 22197067

[14] Delextrat A, Baker J, Cohen DD, Clarke ND. Effect of a simulated soccer match on the functional hamstrings-to-quadriceps ratio in amateur female players. Scand J Med Sci Sports. 2013;23(4):478–86. doi: 10.1111/j.1600-0838.2011.01415.x 22107131

[15] Delextrat A, Gregory J, Cohen D. The use of the functional H:Q ratio to assess fatigue in soccer. Int J Sports Med. 2010;31(3):192–7. doi: 10.1055/s-0029-1243642 20157872

[16] Delextrat A, Piquet J, Matthews MJ, Cohen DD. Strength-Endurance Training Reduces the Hamstrings Strength Decline Following Simulated Football Competition in Female Players. Front Physiol. 2018;9:1059. doi: 10.3389/fphys.2018.01059 30245633 PMC6138075

[17] Grazioli R, Lopez P, Andersen LL, Machado CLF, Pinto MD, Cadore EL et al. Hamstring rate of torque development is more affected than maximal voluntary contraction after a professional soccer match. Eur J Sport Sci. 2019;19(10):1336–41. doi: 10.1080/17461391.2019.1620863 31099729

[18] Greco CC, da Silva WL, Camarda SR, Denadai BS. Fatigue and rapid hamstring/quadriceps force capacity in professional soccer players. Clin Physiol Funct Imaging. 2013;33(1):18–23. doi: 10.1111/j.1475-097X.2012.01160.x 23216761

[19] Greig M, Siegler JC. Soccer-specific fatigue and eccentric hamstrings muscle strength. J Athl Train. 2009;44(2):180–4. doi: 10.4085/1062-6050-44.2.180 19295963 PMC2657020

[20] Jones RI, Ryan B, Todd AI. Muscle fatigue induced by a soccer match-play simulation in amateur Black South African players. J Sports Sci. 2015;33(12):1305–11. doi: 10.1080/02640414.2015.1022572 25764064

[21] Kakavas G, Malliaropoulos N, Kaliakmanis A, Georgios B, Maffulli N. A ninety-minute football match increases hamstring flexibility in professional players. J Biol Regul Homeost Agents. 2020;34(5 Suppl. 1):87–92. IORS Special Issue on Orthopedics. 33739011

[22] Lehnert M, De Ste Croix M, Zaatar A, Hughes J, Varekova R, Lastovicka O. Muscular and neuromuscular control following soccer-specific exercise in male youth: Changes in injury risk mechanisms. Scand J Med Sci Sports. 2017;27(9):975–82. doi: 10.1111/sms.12705 27283749

[23] Lehnert M, De Ste Croix M, Zaatar A, Lipinska P, Stastny P. Effect of a Simulated Match on Lower Limb Neuromuscular Performance in Youth Footballers-A Two Year Longitudinal Study. Int J Environ Res Public Health. 2020;17(22). doi: 10.3390/ijerph17228579 33227935 PMC7699215

[24] Marshall PW, Lovell R, Jeppesen GK, Andersen K, Siegler JC. Hamstring muscle fatigue and central motor output during a simulated soccer match. PLoS One. 2014;9(7):e102753. doi: 10.1371/journal.pone.0102753 25047547 PMC4105441

[25] Marshall PW, Lovell R, Siegler JC. Changes in Passive Tension of the Hamstring Muscles During a Simulated Soccer Match. Int J Sports Physiol Perform. 2016;11(5):594–601. doi: 10.1123/ijspp.2015-0009 26458020

[26] Rahnama N, Lees A, Reilly T. Electromyography of selected lower-limb muscles fatigued by exercise at the intensity of soccer match-play. J Electromyogr Kinesiol. 2006;16(3):257–63. doi: 10.1016/j.jelekin.2005.07.011 16146698

[27] Rahnama N, Reilly T, Lees A, Graham-Smith P. Muscle fatigue induced by exercise simulating the work rate of competitive soccer. J Sports Sci. 2003;21(11):933–42. doi: 10.1080/0264041031000140428 14626373

[28] Small K, McNaughton L, Greig M, Lovell R. Effect of timing of eccentric hamstring strengthening exercises during soccer training: implications for muscle fatigability. J Strength Cond Res. 2009;23(4):1077–83. doi: 10.1519/JSC.0b013e318194df5c 19528859

[29] Small K, McNaughton L, Greig M, Lovell R. The effects of multidirectional soccer-specific fatigue on markers of hamstring injury risk. J Sci Med Sport. 2010;13(1):120–5. doi: 10.1016/j.jsams.2008.08.005 18976956

[30] Small K, McNaughton LR, Greig M, Lohkamp M, Lovell R. Soccer fatigue, sprinting and hamstring injury risk. Int J Sports Med. 2009;30(8):573–8. doi: 10.1055/s-0029-1202822 19455478

[31] Wollin M, Thorborg K, Pizzari T. The acute effect of match play on hamstring strength and lower limb flexibility in elite youth football players. Scand J Med Sci Sports. 2017;27(3):282–8. doi: 10.1111/sms.12655 26926311

[32] Clark RA. Hamstring injuries: risk assessment and injury prevention. Ann Acad Med Singap. 2008;37(4):341–6. 18461220

[33] Green B, Bourne MN, van Dyk N, Pizzari T. Recalibrating the risk of hamstring strain injury (HSI): A 2020 systematic review and meta-analysis of risk factors for index and recurrent hamstring strain injury in sport. British Journal of Sports Medicine. 2020;54(18):1081. doi: 10.1136/bjsports-2019-100983 32299793

[34] Opar DA, Williams MD, Shield AJ. Hamstring strain injuries: factors that lead to injury and re-injury. Sports Med. 2012;42(3):209–26. doi: 10.2165/11594800-000000000-00000 22239734

[35] Arnason A, Sigurdsson SB, Gudmundsson A, Holme I, Engebretsen L, Bahr R. Risk factors for injuries in football. The American journal of sports medicine. 2004;32(1_suppl):5–16. 10.1177/0363546503258912 14754854

[36] Dauty M, Menu P, Fouasson‐Chailloux A. Cutoffs of isokinetic strength ratio and hamstring strain prediction in professional soccer players. Scandinavian journal of medicine & science in sports. 2018;28(1):276–81. doi: 10.1111/sms.12890 28378465

[37] Engebretsen AH, Myklebust G, Holme I, Engebretsen L, Bahr R. Intrinsic risk factors for hamstring injuries among male soccer players: a prospective cohort study. The American journal of sports medicine. 2010;38(6):1147–53. doi: 10.1177/0363546509358381 20335507

[38] Fousekis K, Tsepis E, Poulmedis P, Athanasopoulos S, Vagenas G. Intrinsic risk factors of non-contact quadriceps and hamstring strains in soccer: a prospective study of 100 professional players. British journal of sports medicine. 2011;45(9):709–14. doi: 10.1136/bjsm.2010.077560 21119022

[39] Henderson G, Barnes CA, Portas MD. Factors associated with increased propensity for hamstring injury in English Premier League soccer players. Journal of Science and Medicine in Sport. 2010;13(4):397–402. doi: 10.1016/j.jsams.2009.08.003 19800844

[40] Timmins RG, Bourne MN, Shield AJ, Williams MD, Lorenzen C, Opar DA. Short biceps femoris fascicles and eccentric knee flexor weakness increase the risk of hamstring injury in elite football (soccer): a prospective cohort study. Br J Sports Med. 2016;50(24):1524–35. doi: 10.1136/bjsports-2015-095362 26675089

[41] Bourne MN, Opar DA, Williams MD, Shield AJ. Eccentric knee flexor strength and risk of hamstring injuries in rugby union: a prospective study. The American journal of sports medicine. 2015;43(11):2663–70. doi: 10.1177/0363546515599633 26337245

[42] Gabbe BJ, Bennell KL, Finch CF, Wajswelner H, Orchard J. Predictors of hamstring injury at the elite level of Australian football. Scandinavian journal of medicine & science in sports. 2006;16(1):7–13. doi: 10.1111/j.1600-0838.2005.00441.x 16430675

[43] Opar D, Williams M, Timmins R, Hickey J, Duhig S, Shield A. Eccentric hamstring strength and hamstring injury risk in Australian footballers. Medicine and Science in Sports and Exercise. 2015;47(4):857–65. doi: 10.1249/MSS.0000000000000465 25137368

[44] Roe M, Murphy JC, Gissane C, Blake C. Hamstring injuries in elite Gaelic football: an 8-year investigation to identify injury rates, time-loss patterns and players at increased risk. British journal of sports medicine. 2018;52(15):982–8. doi: 10.1136/bjsports-2016-096401 27797729

[45] De Vos R-J, Reurink G, Goudswaard G-J, Moen MH, Weir A, Tol JL. Clinical findings just after return to play predict hamstring re-injury, but baseline MRI findings do not. British journal of sports medicine. 2014;48(18):1377–84. doi: 10.1136/bjsports-2014-093737 25037201

[46] Freckleton G, Cook J, Pizzari T. The predictive validity of a single leg bridge test for hamstring injuries in Australian Rules Football Players. British journal of sports medicine. 2014;48(8):713–7. doi: 10.1136/bjsports-2013-092356 23918443

[47] Goossens L, Witvrouw E, Vanden Bossche L, De Clercq D. Lower eccentric hamstring strength and single leg hop for distance predict hamstring injury in PETE students. European journal of sport science. 2015;15(5):436–42. doi: 10.1080/17461391.2014.955127 25189278

[48] Schuermans J, Van Tiggelen D, Danneels L, Witvrouw E. Susceptibility to hamstring injuries in soccer: a prospective study using muscle functional magnetic resonance imaging. The American journal of sports medicine. 2016;44(5):1276–85. doi: 10.1177/0363546515626538 26912281

[49] Watsford ML, Murphy AJ, McLachlan KA, Bryant AL, Cameron ML, Crossley KM et al. A prospective study of the relationship between lower body stiffness and hamstring injury in professional Australian rules footballers. Am J Sports Med. 2010;38(10):2058–64. doi: 10.1177/0363546510370197 20595555

[50] Silva JR, Rumpf MC, Hertzog M, Castagna C, Farooq A, Girard O et al. Acute and Residual Soccer Match-Related Fatigue: A Systematic Review and Meta-analysis. Sports Med. 2018;48(3):539–83. doi: 10.1007/s40279-017-0798-8 29098658

[51] da Silva C, Lovell R. External Validity of the T-SAFT90: A Soccer Simulation Including Technical and Jumping Activities. International Journal of Sports Physiology and Performance. 2020 (corrected in 2022);15(8):1074–80. doi: 10.1123/ijspp.2019-0057 32814311

[52] Liu H, Garrett WE, Moorman CT, Yu B. Injury rate, mechanism, and risk factors of hamstring strain injuries in sports: A review of the literature. Journal of Sport and Health Science. 2012;1(2):92–101. doi: 10.1016/j.jshs.2012.07.003

[53] Kenneally-Dabrowski CJB, Brown NAT, Lai AKM, Perriman D, Spratford W, Serpell BG. Late swing or early stance? A narrative review of hamstring injury mechanisms during high-speed running. Scand J Med Sci Sports. 2019;29(8):1083–91. doi: 10.1111/sms.13437 31033024

[54] Chumanov ES, Heiderscheit BC, Thelen DG. Hamstring musculotendon dynamics during stance and swing phases of high-speed running. Med Sci Sports Exerc. 2011;43(3):525–32. doi: 10.1249/MSS.0b013e3181f23fe8 20689454 PMC3057086

[55] Kujala UM, Orava S, Järvinen M. Hamstring injuries. Current trends in treatment and prevention. Sports medicine (Auckland, NZ). 1997;23(6):397–404. doi: 10.2165/00007256-199723060-00005 9219322

[56] Thelen D, Chumanov E, Hoerth D, Best T, Swanson S, Li L et al. Hamstring Muscle Kinematics during Treadmill Sprinting. Medicine and science in sports and exercise. 2005;37:108–14. doi: 10.1249/01.mss.0000150078.79120.c8 15632676

[57] Roig Pull M, Ranson C. Eccentric muscle actions: Implications for injury prevention and rehabilitation. Physical Therapy in Sport. 2007;8(2):88–97. doi: 10.1016/j.ptsp.2006.11.005

[58] Raiteri BJ, Beller R, Hahn D. Biceps Femoris Long Head Muscle Fascicles Actively Lengthen During the Nordic Hamstring Exercise. Front Sports Act Living. 2021;3:669813. doi: 10.3389/fspor.2021.669813 34179775 PMC8219857

[59] Barrett S, Guard A, Lovell R. SAFT90 simulates the internal and external loads of competitive soccer match-play. 2013. p. 95–100.

[60] Faul F, Erdfelder E, Buchner A, Lang A-G. Statistical power analyses using G*Power 3.1: Tests for correlation and regression analyses. Behavior Research Methods. 2009;41(4):1149–60. doi: 10.3758/BRM.41.4.1149 19897823

[61] Franchi MV, Fitze DP, Raiteri BJ, Hahn D, Spörri J. Ultrasound-derived Biceps Femoris Long Head Fascicle Length: Extrapolation Pitfalls. Med Sci Sports Exerc. 2020;52(1):233–43. doi: 10.1249/MSS.0000000000002123 31403609

[62] Bujang MA, Baharum N, editors. A simplified guide to determination of sample size requirements for estimating the value of intraclass correlation coefficient: a review2017.

[63] Gibbons T, Bird M-L. Exercising on Different Unstable Surfaces Increases Core Abdominal Muscle Thickness; An Observational Study Using Real Time Ultrasound. Journal of Sport Rehabilitation. 2018;28:1–20. doi: 10.1123/jsr.2017-0385 30526226

[64] Craig CL, Marshall AL, Sjöström M, Bauman AE, Booth ML, Ainsworth BE et al. International physical activity questionnaire: 12-country reliability and validity. Med Sci Sports Exerc. 2003;35(8):1381–95. doi: 10.1249/01.MSS.0000078924.61453.FB 12900694

[65] Lovell R, Knapper B, Small K, editors. Physiological responses to SAFT90: a new soccer-specific match simulation. Verona-Ghirada Team Sports Conference Proceedings; 2008.

[66] Timmins RG, Shield AJ, Williams MD, Lorenzen C, Opar DA. Biceps femoris long head architecture: a reliability and retrospective injury study. Med Sci Sports Exerc. 2015;47(5):905–13. doi: 10.1249/MSS.0000000000000507 25207929

[67] Klimstra M, Dowling J, Durkin JL, MacDonald M. The effect of ultrasound probe orientation on muscle architecture measurement. Journal of Electromyography and Kinesiology. 2007;17(4):504–14. doi: 10.1016/j.jelekin.2006.04.011 16919969

[68] Potier TG, Alexander CM, Seynnes OR. Effects of eccentric strength training on biceps femoris muscle architecture and knee joint range of movement. Eur J Appl Physiol. 2009;105(6):939–44. doi: 10.1007/s00421-008-0980-7 19271232

[69] Nimphius S, McGuigan MR, Newton RU. Changes in muscle architecture and performance during a competitive season in female softball players. J Strength Cond Res. 2012;26(10):2655–66. doi: 10.1519/JSC.0b013e318269f81e 22847524

[70] Blazevich A, Cannavan D, Coleman D, Msc S. Influence of concentric and eccentric resistance training on architectural adaptation in human quadriceps muscles. Journal of applied physiology (Bethesda, Md: 1985). 2007;103:1565–75. doi: 10.1152/japplphysiol.00578.2007 17717119

[71] Gerlinger M, Horswell S, Larkin J, Rowan AJ, Salm MP, Varela I et al. Genomic architecture and evolution of clear cell renal cell carcinomas defined by multiregion sequencing. Nat Genet. 2014;46(3):225–33. doi: 10.1038/ng.2891 24487277 PMC4636053

[72] Seri B, García-Verdugo JM, Collado-Morente L, McEwen BS, Alvarez-Buylla A. Cell types, lineage, and architecture of the germinal zone in the adult dentate gyrus. J Comp Neurol. 2004;478(4):359–78. doi: 10.1002/cne.20288 15384070

[73] Hallet P. A reliability study examining the inter-and intra-observer reliability of the muscle capacity tests included in the ECB musculoskeletal screening protocol [Masters]. University of Nottingham. 2010.

[74] Rey E, Paz-Domínguez Á, Porcel-Almendral D, Paredes-Hernández V, Barcala-Furelos R, Abelairas-Gómez C. Effects of a 10-Week Nordic Hamstring Exercise and Russian Belt Training on Posterior Lower-Limb Muscle Strength in Elite Junior Soccer Players. J Strength Cond Res. 2017;31(5):1198–205. doi: 10.1519/JSC.0000000000001579 27467517

[75] Munro AG, Herrington LC. Between-session reliability of four hop tests and the agility T-test. J Strength Cond Res. 2011;25(5):1470–7. doi: 10.1519/JSC.0b013e3181d83335 21116200

[76] Weir JP. Quantifying test-retest reliability using the intraclass correlation coefficient and the SEM. J Strength Cond Res. 2005;19(1):231–40. doi: 10.1519/15184.1 15705040

[77] Portney LG, Watkins MP. Foundations of clinical research: applications to practice. Pearson/Prentice Hall Upper Saddle River, NJ; 2009.

[78] Borenstein M, Rothstein H, Cohen J. Comprehensive meta-analysis: A computer program for research synthesis. Englewood, NJ: Biostat; 1999.

[79] Lakens D. Calculating and reporting effect sizes to facilitate cumulative science: a practical primer for t-tests and ANOVAs. Frontiers in Psychology. 2013;4(863). doi: 10.3389/fpsyg.2013.00863 24324449 PMC3840331

[80] Brydges CR. Effect Size Guidelines, Sample Size Calculations, and Statistical Power in Gerontology. Innov Aging. 2019;3(4):igz036-igz. doi: 10.1093/geroni/igz036 31528719 PMC6736231

[81] Pincheira PA, Boswell MA, Franchi MV, Delp SL, Lichtwark GA. Biceps femoris long head sarcomere and fascicle length adaptations after 3 weeks of eccentric exercise training. Journal of Sport and Health Science. 2021. doi: 10.1016/j.jshs.2021.09.002 34509714 PMC8847943

[82] Seymore KD, Domire ZJ, DeVita P, Rider PM, Kulas AS. The effect of Nordic hamstring strength training on muscle architecture, stiffness, and strength. Eur J Appl Physiol. 2017;117(5):943–53. doi: 10.1007/s00421-017-3583-3 28280975

[83] Wiesinger HP, Scharinger M, Kösters A, Gressenbauer C, Müller E. Specificity of eccentric hamstring training and the lack of consistency between strength assessments using conventional test devices. Sci Rep. 2021;11(1):13417. doi: 10.1038/s41598-021-92929-y 34183742 PMC8239011

[84] Yagiz G, Akaras E, Kubis H-P, Owen JA. Heterogeneous effects of eccentric training and nordic hamstring exercise on the biceps femoris fascicle length based on ultrasound assessment and extrapolation methods: A systematic review of randomised controlled trials with meta-analyses. PLOS ONE. 2021;16(11):e0259821. doi: 10.1371/journal.pone.0259821 34752499 PMC8577763

[85] Goncçalves BAM. How does a simulated soccer match affect regional differences in biceps femoris muscle architecture? [master’s thesis]. Jyväskylä [Finland]: University of Jyväskylä; 2017.

