# Supplementary material for: Alterations in biceps femoris long head fascicle length, Eccentric hamstring strength qualities and single-leg hop distance throughout the ninety minutes of TSAFT90 simulated football match
Source: PLoS One. 2022 Dec 9;17(12):e0278222. doi: 10.1371/journal.pone.0278222 (PMC9733901; doi:10.1371/journal.pone.0278222)
Supplement: S1 File — (PDF) [file pone.0278222.s001.pdf]

1. Participants' Physical Features

Player	Age	Height	Weight
1.00	34.00	169.00	83.20
2.00	19.00	185.50	61.60
3.00	21.00	170.50	70.60
4.00	32.00	171.50	72.60
5.00	19.00	171.00	77.70
6.00	39.00	171.20	77.40
7.00	23.00	175.30	75.80
8.00	25.00	172.50	71.90
9.00	32.00	170.00	77.90
10.00	23.00	165.90	67.40
11.00	27.00	177.60	54.40
12.00	25.00	167.00	68.40
13.00	22.00	166.50	73.90
14.00	25.00	176.80	77.80
15.00	20.00	176.50	73.40

2. Single-Leg Hamstring Bridge Test Results

Player	D m1	D m2	ND m1	ND m2	AV m1	Av m2
1.00	30.00	20.00	25.00	19.00	27.50	19.50
2.00	31.00	13.00	31.00	17.00	31.00	15.00
3.00	40.00	30.00	34.00	30.00	37.00	30.00
4.00	18.00	12.00	16.00	12.00	17.00	12.00
5.00	23.00	15.00	22.00	12.00	22.50	13.50

6.00	23.00	16.00	21.00	15.00	22.00	15.50
7.00	25.00	10.00	25.00	10.00	25.00	10.00
8.00	29.00	18.00	27.00	17.00	28.00	17.50
9.00		DROP OUT				
10.00	28.00	19.00	25.00	17.00	26.50	18.00
11.00	34.00	20.00	30.00	20.00	32.00	20.00
12.00		DROP OUT				
13.00	23.00	20.00	25.00	19.00	24.00	19.50
14.00	33.00	20.00	32.00	20.00	32.50	20.00
15.00	40.00	30.00	41.00	30.00	40.50	30.00
16.00	31.00	26.00	24.00	23.00	27.50	24.50
17.00	35.00	36.00	31.00	33.00	33.00	34.50

Abbreviations: AV, the average of the legs; D, dominant leg; m, measurement; ND, non-dominant leg.

3. Maximal Eccentric Hamstring Strength

Player	D m1	D m2	ND m1	ND m2	AV m1	AV m2
1.00	250.00	178.00	230.00	172.00	240.00	175.00
2.00	216.00	178.00	208.00	168.00	212.00	173.00
3.00	208.00	160.00	192.00	138.00	200.00	149.00
4.00	172.00	140.00	162.00	134.00	167.00	137.00
5.00	250.00	226.00	230.00	192.00	240.00	209.00
6.00	208.00	116.00	198.00	118.00	203.00	117.00
7.00	194.00	160.00	176.00	114.00	185.00	137.00
8.00	192.00	140.00	184.00	142.00	188.00	141.00
9.00		DROP OUT				

10.00	188.00	176.00	178.00	150.00	183.00	163.00
11.00	238.00	182.00	196.00	174.00	217.00	178.00
12.00		DROP OUT				
13.00	230.00	192.00	220.00	190.00	225.00	191.00
14.00	218.00	128.00	214.00	114.00	216.00	121.00
15.00	228.00	244.00	224.00	240.00	226.00	242.00
16.00	248.00	206.00	238.00	178.00	243.00	192.00
17.00	252.00	266.00	250.00	264.00	251.00	265.00

Abbreviations: AV, the average of the legs; D, dominant leg; m, measurement; ND, non-dominant leg.

4. Maximal Eccentric Hamstring Strength Reliability

Player	D m1	D m2	ND m1	ND m2
1.00	250.00	238.00	230.00	210.00
2.00	200.00	216.00	208.00	206.00
3.00	196.00	208.00	178.00	192.00
4.00	172.00	166.00	162.00	148.00
5.00	232.00	250.00	216.00	230.00
6.00	206.00	208.00	190.00	198.00
7.00	194.00	192.00	176.00	168.00
8.00	188.00	192.00	182.00	184.00
9.00	286.00	290.00	262.00	256.00
10.00	186.00	188.00	174.00	178.00
11.00	236.00	238.00	190.00	196.00
12.00	200.00	202.00	170.00	164.00
13.00	230.00	230.00	220.00	220.00

14.00	216.00	218.00	214.00	208.00
15.00	228.00	224.00	222.00	226.00
16.00	248.00	224.00	238.00	226.00
17.00	252.00	250.00	244.00	250.00

Abbreviations: D, dominant leg; m, measurement; ND, non-dominant leg.

5. The average heart rate as a percentage of maximum

Player	m1	m2	m3	m4	m5	m6
1	66.00	63.00	68.00	74.00	77.00	77.00
2	72.00	72.00	76.00	74.00	86.00	82.00
3	83.00	81.00	79.00	79.00	79.00	85.00
4	68.00	72.00	69.00	71.00	74.00	78.00
5	81.00	76.00	73.00	68.00	73.00	83.00
6	75.00	84.00	80.00	84.00	81.00	83.00
7	77.00	82.00	78.00	78.00	80.00	81.00
8	86.00	83.00	80.00	80.00	80.00	87.00
9	77.00	78.00	72.00	64.00	72.00	79.00
10	73.00	72.00	69.00	63.00	63.00	76.00
11	79.00	75.00	77.00	68.00	71.00	77.00
12	71.00	67.00	66.00	65.00	66.00	71.00
13	81.00	80.00	78.00	75.00	81.00	94.00
14	86.00	89.00	88.00	90.00	91.00	98.00

Abbreviations: m, measurement.

6. Biceps Femoris Long Head Fascicle Length

PLAYER	DBASELINE	DHALFTIME	DFULLTIME	NDBASELINE	NDHALFTIME	NDFULLTIME	AVBASELINE	AVHALFTIME	AVFULLTIME
1	7.976207	8.168804	7.299304	7.974163	8.045174	7.395348	7.975185	8.106989	7.347326
2	8.690913	7.74938	8.186685	7.626772	7.368272	7.371337	8.158842	7.558826	7.779011
3	8.193326	6.981033	8.307761	8.143261	8.831402	8.084511	8.168293	7.906217	8.196136
4	8.498826	10.00334	9.874087	8.271489	9.425543	7.642098	8.385158	9.71444	8.758092
5	12.31502	11.78321	13.06191	12.2108	13.20087	11.66468	12.26291	12.49204	12.3633
6	11.79393	11.21665	10.81664	8.432924	7.789228	9.78162	10.11343	9.50294	10.29913
7	9.140989	9.697837	8.770609	9.519543	10.11675	9.61763	9.330266	9.907293	9.19412
8	7.463804	8.77112	7.93738	6.818576	7.029054	7.664576	7.14119	7.900087	7.800978
9	DROP OUT								
10	6.875793	7.970587	7.962413	7.494457	9.393359	9.630402	7.185125	8.681973	8.796408
11	10.16528	11.22636	9.857228	9.032685	7.814772	8.661283	9.598984	9.520565	9.259255
12	DROP OUT								
13	10.48049	10.20922	9.404598	11.67899	10.61638	10.89634	11.07974	10.4128	10.15047
14	10.7773	10.5137	12.26036	11.25343	10.65214	11.52062	11.01537	10.58292	11.89049
15	11.95333	11.35765	11.74847	10.99136	10.43655	10.65725	11.47234	10.8971	11.20286
16	10.71907	10.37014	11.045	11.50172	11.14411	11.65855	11.11039	10.75713	11.35178
17	10.27665	10.44728	10.72366	10.91984	11.06595	11.25548	10.59824	10.75661	10.98957

Abbreviations: D, dominant leg; ND, non-dominant leg.

7. Fascicle length Reliability

PLAYER	DDAY 1	DBASELINE	NDDAY 1	NDBASELINE
1	7.797913	7.976207	7.670707	7.974163
2	8.73638	8.690913	7.629837	7.626772
3	8.175446	8.193326	8.341989	8.143261
4	8.572391	8.498826	8.075826	8.271489
5	12.31553	12.31502	12.3799	12.2108
6	11.3975	11.79393	8.661283	8.432924
7	8.967293	9.140989	9.077641	9.519543

8	8.071739	7.463804	6.818065	6.818576
9	DROP OUT			
10	6.546793	6.875793	7.292152	7.494457
11	9.627848	10.16528	9.020424	9.032685
12	DROP OUT			
13	10.90911	10.48049	11.12878	11.67899
14	10.46976	10.7773	11.24782	11.25343
15	11.98245	11.95333	10.68433	10.99136
16	11.16812	10.71907	10.9653	11.50172
17	10.65623	10.27665	11.23862	10.91984

Abbreviations: D, dominant leg; ND, non-dominant leg.

8. Single-leg Hop Distance

PLAYER	DBASELINE	DHALFTIME	DFULLTIME	NDBASELINE	NDHALFTIME	NDFULLTIME	AVBASELINE	AVHALFTIME	AVFULLTIME
ASF1	172	177	173	177	180	180	174.5	178.5	176.5
ASF2	232	236	231	227	227	235	229.5	231.5	233
ASF3	144	158	146	142	135	139	143	146.5	142.5
ASF4	148	169	171	175	183	181	161.5	170	176
ASF5	145	176	172	167	158	173	156	167	172.5
ASF6	184	186	185	171	163	173	177.5	174.5	179
ASF7	168	177	177	165	156	140	166.5	166.5	158.5
ASF8	199	188	192	183	179	159	191	183.5	175.5
ASF9	DROP OUT								
ASF10	167	182	188	163	172	166	165	177	177
ASF11	187	201	201	173	197	175	180	199	188
ASF12	DROP OUT								
ASF13	213	204	225	195	191	198	204	197.5	211.5
ASF14	181	168	168	186	171	180	183.5	169.5	174
ASF15	195	186	191	191	176.9	188	193	181.45	189.5

ASF16	228	219	218	223.7	213	213.5	225.8	216	215.75
ASF17	197	190	188	161.5	200.2	187	179.25	195.1	187.5

Abbreviations: D, dominant leg; ND, non-dominant leg.
